# Supplementary material for: Automated measurement of cardiomyocyte monolayer contraction using the Exeter Multiscope
Source: Biomed Opt Express. 2025 Oct 29;16(11):4716–29. doi: 10.1364/BOE.578208 (PMC12642980; doi:10.1364/BOE.578208)
Supplement: Supplement 1 [file boe-16-11-4716-s001.pdf]

Supplemental Document

Biomedical Optics EXPRESS

Automated measurement of cardiomyocyte monolayer contraction using the Exeter Multiscope: supplement

SHARIKA MOHANAN,[1] DAVID HORSELL,[2] TAYLOR WATTERS,[3] MOHAMMADREZA GHASEMI,[3] LEWIS HENDERSON,[3] CAROLINE MÜLLENBROICH,[1] GIL BUB,[4] FRANCIS BURTON,[5] GODFREY SMITH,[5] AND ALEXANDER D. CORBETT[2,6,*]

[1]*School of Physics and Astronomy, University of Glasgow, G12 8SU, UK*
[2]*Department of Physics and Astronomy, University of Exeter, EX4 4QL, UK*
[3]*Clyde Biosciences, BioCity Scotland, ML1 5UH, UK*
[4]*Department of Physiology, McGill University, Montréal, H3G 1Y66, Canada*
[5]*School of Cardiovascular & Metabolic Health, University of Glasgow, G12 8TA, UK*
[6]*Living Systems Institute, University of Exeter, EX4 4QD, UK*
[*]*a.corbett@exeter.ac.uk*

This supplement published with Optica Publishing Group on 29 October 2025 by The Authors under the terms of the Creative Commons Attribution 4.0 License in the format provided by the authors and unedited. Further distribution of this work must maintain attribution to the author(s) and the published article's title, journal citation, and DOI.

Supplement DOI: https://doi.org/10.6084/m9.figshare.30434092

Parent Article DOI: https://doi.org/10.1364/BOE.578208

AUTOMATED MEASUREMENT OF CARDIOMYOCYTE MONOLAYER CONTRACTION USING THE EXETER MULTISCOPE: SUPPLEMENTAL DOCUMENT

S1: Optimising Multiscope analysis parameters

Before processing the MS image data, three parameters first needed to be determined: the frame delay, window size and focal depth. The frame delay determines the time interval over which the raw image data are processed (Figure 3). For the CB data, this was set at around 20% of the interval between contraction. For the MS data, there are approximately 10 sampling points between peaks, meaning the frame delay is expected to be around 2 frames. The optimal frame delay is that which maximises the signal to background ratio of the peaks in the contraction speed trace. The signal to background ratio was calculated as the ratio of the highest 10% of values in the trace to the lowest 10% of values in the trace. The same data set, composed of all nine wells, was processed with a 1-, 2-, 3-, 5-, and 10-frame delay. The average signal to background ratio was calculated for all frame delays. The results are shown in Figure 10(A), which indicates that there is a marginal advantage to using a 2- or 3-frame delay. In this case it was decided to use a 2-frame delay (= 0.54 s) for all MS data calculations.

Second, the illumination wavelength that produced the best sample focus was determined by using the SBR as before, but this time comparing the three colour channels for all nine wells. The results indicated that the focal plane of the red channel was closest to the monolayer sample, which corresponds well with a visual inspection of the data from each channel.

Finally, the width of the region of interest used to crop the raw image data from each well was determined. This was calculated for window half-widths of 25, 50, 80, 100 and 150 pixels. The main trade off here was ensuring that the window was large enough to maximise the signal contained within the processed frame, whilst being small enough to avoid any effects from the edges of the well. In this analysis, the first well (well #44) had to be removed from this analysis due to the presence of a bubble which interfered with the average calculation.

S2: Mathematical comparison of PV and MM algorithms

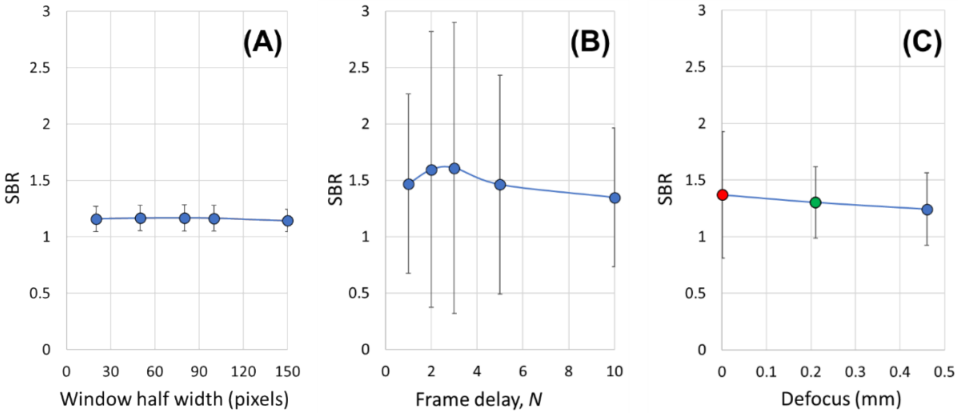

FigureS1: Variation in contraction signal contrast as a function of cropping window size (A), frame delay and (C) illumination wavelength.

This section compares the MM and PV algorithms in terms of the noise propagation. This is important to understand how differences in these output metrics originate and how they scale with the size of the frame delay, N. In this analysis we consider a single pixel value, x_i, corresponding to frame i (see Figure 3). For brightfield imaging, we assume that the photon

statistics are dominated by shot noise (Poisson distribution), whereby the pixel values recorded on the camera are taken from a distribution with mean and variance values equal to x_i.

Both MM and PV algorithms quantify the change in pixel value over a pre-defined frame delay (*N* frames). The MM metric calculates the modulus of the difference, whereas the PV calculates the standard deviation of the pixel values over the interval. We want to identify how the errors in x_i propagate to the metric used in each case.

Beginning with the MM metric, we define this as:

$$f_{MM} = |x_{N+i} - x_i| \qquad \text{(S1)}$$

For which the error is propagated as:

$$\sigma^2_{f_{MM}} = \sigma^2_{x_{N+i}} + \sigma^2_{x_i} \qquad \text{(S2)}$$

Which, if the pixel values are close to each other ($x_{N+i} \approx x_i$) we can simplify to:

$$\sigma^2_{f_{MM}} = 2x_i \qquad \text{(S3)}$$

For the PV metric, we begin by calculating the variance (f_{VAR}) of the pixel values over time:

$$f_{VAR} = \frac{1}{N}\left[(x_i - \mu)^2 + (x_{i+1} - \mu)^2 + \ldots(x_{N+i} - \mu)^2\right] \qquad \text{(S4)}$$

Where μ is the mean pixel value over the range x_i to x_{N+i}. We employ the error propagation formula:

$$\sigma^2_{f_{VAR}} = \left(\frac{df_{VAR}}{dx_i}\right)^2 \sigma^2_{x_i} + \left(\frac{df_{VAR}}{dx_{i+1}}\right)^2 \sigma^2_{x_{i+1}} + \ldots \left(\frac{df_{VAR}}{dx_{N+i}}\right)^2 \sigma^2_{x_{N+i}} \qquad \text{(S5)}$$

Where the partial derivative in each of these terms has the form:

$$\frac{df_{VAR}}{dx_i} = \frac{2}{N}(x_i - \mu) \qquad \text{(S6)}$$

Making the same assumption that the pixel values are not very different between frames, we can write:

$$\sigma^2_{f_{VAR}} = \left[\frac{2}{N}(x_i - \mu)\right]^2 \sigma^2_{x_i} + \left[\frac{2}{N}(x_{i+1} - \mu)\right]^2 \sigma^2_{x_i} + \ldots \left[\frac{2}{N}(x_{N+i} - \mu)\right]^2 \sigma^2_{x_i} \qquad \text{(S7)}$$

$$= \left(\frac{4}{N}\right)\sigma^2_{x_i} \sum_i^{N+i} \frac{(x_j - \mu)^2}{N} = \left(\frac{4}{N}\right)\sigma^2_{x_i} f_{VAR} = \left(\frac{4}{N}\right) x_i\, f_{VAR} \qquad \text{(S8)}$$

Finally, we can relate the error in the pixel variance, $\sigma^2_{f_{VAR}}$, to the error in the standard deviation of the pixel values, $\sigma^2_{f_{STD}}$. Given that $f_{VAR} = f^2_{STD}$, we can write:

$$\sigma^2_{f_{VAR}} = \left(\frac{df_{VAR}}{df_{STD}}\right)^2 \sigma^2_{f_{STD}} = (2f_{STD})^2 \sigma^2_{f_{STD}} \qquad \text{(S9)}$$

Making f_{STD} the focus of this equation we have:

$$\sigma_{f_{STD}} = \frac{\sigma_{f_{VAR}}}{(2f_{STD})} = \frac{\left(\frac{2}{\sqrt{N}}\right)\sigma_{x_i} f_{STD}}{(2f_{STD})} = \frac{\sigma_{x_i}}{\sqrt{N}} = \sqrt{\frac{x_i}{N}} \qquad \text{(S10)}$$

We can then write down the ratio of the standard error for these two metrics as:

$$\sigma_{f_{STD}} \big/ \sigma_{f_{MM}} = \frac{1}{\sqrt{2N}} \qquad \text{(S11)}$$

This predicts a 2X reduction in noise in the PV metric for the MS data (N=2) and a 6.3X reduction in noise for the CB data (N=20) relative to the MM metric. Whilst the improvements are harder to see in the MS data, there are clear differences in the CB data between the MM (green) and PV (blue) traces (Figure 8).
